# Identification of Patients with Pentosan Polysulfate Sodium-Associated Maculopathy through Screening of the Electronic Medical Record at an Academic Center

**DOI:** 10.1155/2020/8866961

**Published:** 2020-12-17

**Authors:** Kendall Higgins, R. Joel Welch, Colin Bacorn, Glenn Yiu, Jennifer Rothschild, Susanna S. Park, Ala Moshiri

**Affiliations:** ^1^Department of Ophthalmology & Vision Science, School of Medicine, University of California Davis, Sacramento, CA, USA; ^2^The University of Miami, Leonard M. Miller School of Medicine, Miami, FL, USA; ^3^Department of Urologic Surgery, School of Medicine, University of California Davis, Sacramento, CA, USA

## Abstract

**Aims:**

This chart review of a quaternary academic medical center electronic medical record (EMR) aimed to identify patients at risk of development of maculopathy with exposure to pentosan polysulfate sodium (PPS).

**Methods:**

A review of electronic medical records of a quaternary medical center of patients with either documented exposure to PPS or diagnosis of interstitial cystitis (IC) from 2007 to 2019 was performed for retinal imaging and visual acuity; the study was conducted in August of 2019.

**Results:**

216 charts were included for analysis, of which 96 had documented eye exams and 24 had retinal imaging done. We identified three patients with maculopathy in the context of long-term exposure to PPS via chart review, and one additional patient was identified by referral. The median PPS exposure duration was 11 years (range 7 to 19 years). Median logMAR BCVA OD 0.6 range was 0.0–1.9 (approximate Snellen equivalent 20/80 range (20/20–20/1600)) and OS 0.7 range was 0.1–1.9 (approximate Snellen equivalent 20/100 range (20/25–20/1600)). Ultrawidefield color fundus imaging and fundus autofluorescence revealed findings of pigmentary changes and patchy macular atrophy. Optical coherence tomography (OCT) demonstrated outer retinal thinning and increased choroidal transmission coincident with areas of atrophy seen on fundus imaging.

**Conclusions:**

Less than half of patients at risk for development of maculopathy due to exposure to PPS had received eye examinations, suggesting that those at risk are not receiving adequate screening. We found two patients with PPS maculopathy who had relatively preserved central vision, one patient with bitemporal vision loss, and one patient who developed vision loss in both eyes.

## 1. Introduction

Pentosan polysulfate sodium (PPS) is an oral drug used for interstitial cystitis (IC), a chronic urinary pain syndrome thought to be due to irritation of the bladder interstitium. PPS is a heparin-like glycosaminoglycan (GAG) that is proposed to function by binding the luminal bladder epithelium and preventing irritants within the lumen of the bladder from reaching the interstitium [1]. In 2018, a case series of 6 patients demonstrated a possible link between long-term PPS exposure and pigmentary maculopathy [2]. In 2019, a multi-institutional case series identified 35 patients across 4 institutions with long-term exposure to PPS and pigmentary maculopathy [3]. An additional case report of maculopathy after long-term exposure to PPS was found to continue to progress for 6 years after discontinuation of this medication resulting in severe vision loss [4]. In an additional case series, 11 patients with long-term PPS exposure and development of pigmentary maculopathy were followed after cessation of PPS for at least six months. No eyes had a marked improvement in disease following cessation and 9 of these 11 patients reported worsening visual symptoms at the final follow-up [5].

In a retrospective cohort study using the MarketScan database, 49,899 patients with IC diagnosis were divided into those using and those not using PPS and were assessed for the development of maculopathy. There was no difference in the development of maculopathy between groups. In addition, there was no dose-dependent relationship between PPS exposure and diagnosis of maculopathy [6]. Another matched cohort study compared cohorts of PPS users (3,012) versus non-PPS users (15,060) at 5 years of follow-up; it showed no significant risk of developing an atypical maculopathy and/or age-related macular degeneration (AMD). However, at 7 years, comparing PPS users (1,604) and non-PPS users (8,017), the authors found a significantly increased odds ratio of developing an atypical maculopathy and/or AMD [7].

The association between PPS exposure and maculopathies has been reported by other groups [2–7]. Here, we report the results of a screen of the electronic medical record (EMR) at a quaternary academic center to identify additional cases of maculopathy in patients taking PPS and demonstrate the ability of an EMR query to identify patients at risk for development of maculopathy.

## 2. Methods

### 2.1. Consent

This retrospective screen was approved by the UC Davis Institutional Review Board. Information was gathered and secured in compliance with the Health Insurance Portability and Accountability Act. All data were deidentified and shared securely among researchers. This study met all requirements for a large scale screen waiver of informed consent per institutional policy.

### 2.2. Case Identification and Analysis

University of California Davis (UCD) Eye Center and Health System EMR were queried from 2007 to 2019 for either exposure to PPS or diagnosis of IC in August of 2019. EMR of the University of California Davis (UCD) Eye Center and Health System is the Epic electronic health record system (Epic Systems Corporation), version “November 2018” of Hyperspace, was used for individual chart review.

A total of 185 patients had exposure to PPS documented in the medications section of EMR, while 258 patients had a reported diagnosis of IC. Both were searched using either structured or unstructured (free text) data in the medication or diagnosis section of EMR. Of the 258 patients with diagnosis of IC, 127 were already captured by having documented exposure to PPS in the medication section of EMR. The remaining 131 unique patients who did not have documented exposure to PPS in the medication section of EMR and individual notes were reviewed to confirm the history of exposure to PPS. One hundred eighty-five patients with PPS exposure were noted in the medication section of EMR and 131 with a document IC diagnosis but PPS exposure only were documented in individual notes; a total of 316 individual charts with documented exposure to PPS were reviewed. Of these 316 charts, a total of 216 were included in the study. One hundred patients were excluded from the study due to one or more of the following reasons: (1) The only visit was to the emergency department at UC Davis, and therefore they did not have adequate information regarding their exposure to PPS or IC diagnosis. (2) They were prescribed PPS but this could not be corroborated in the notes of the prescribing medical provider. (3) Both their PPS exposure had an unclear history and their diagnosis of IC was then ruled to be another condition.

Each patient chart was reviewed for various demographic and clinical characteristics: age, IC diagnosis, race, gender, PPS cumulative exposure, body mass index (BMI), pack-years smoking, alcohol and drug use, and chronic medical conditions. The chart review feature of EMR was queried for all past prescriptions of PPS. Duration of exposure was considered as the time period the prescription was active. This was confirmed by individual provider notes when available. In each of the three cases highlighted, the exposure histories were documented in the chart review medication section of EMR, confirmed with review of individual provider notes, and confirmed on in-person interview follow-up. One additional case (patient 4) was identified from an outside referral and is included in statistical analysis; however, it was not part of the original chart review. The drug exposure history for the 93 patients without maculopathy may be inaccurate since direct corroboration with these patients was not possible in the way that it was with the affected patients. If available, a summary of the findings from the most recent ophthalmologic exam was obtained including cumulative exposure of PPS at time of examination, most prominent eye symptom, best corrected visual acuity (BVCA), intraocular pressure, cornea status, lens status, and macular diagnosis if present.

### 2.3. Statistics

All data were tabulated on Microsoft Excel 2016 and measures of central tendencies (mean, median, and range) were obtained using built-in functions. An independent two-sample *t*-test was used to assess statistical significance between continuous data with a *P* value of less than 0.05 being considered statistically significant.

## 3. Results

### 3.1. Demographics and Clinical Presentation

The screen for patients with PPS exposure history yielded 316 charts. After applying exclusion criteria, data of 216 charts were included for analysis. Among these 216 patients, 96 patients had a documented ophthalmic examination at the University of California Davis Eye Center (Table 1).

Of these 96 patients, 24 had macular imaging which was then reviewed for the retinal abnormalities noted in the original report [2]: (1) autofluorescent imaging revealing a densely packed array of hyperautofluorescent and hypoautofluorescent spots involving the posterior pole, (2) fundus photography revealing macular hyperpigmented spots, yellow-orange deposits, and/or patchy retinal pigment epithelium (RPE) atrophy, and (3) optical coherence tomography (OCT) imaging demonstrating focal thickening or elevation of the RPE with associated hyperreflectance on near-infrared reflectance imaging.

Five had fundus autofluorescence imaging done, of which 3 had retinal imaging findings consistent with prior reports of maculopathy in the context of long-term PPS exposure [2, 3]. Outside of the chart review, one additional patient was referred (patient 4) and found to have findings consistent with PPS-associated maculopathy (Table 2).

Of the remaining group of 93 patients with PPS exposure who had ophthalmic evaluation but had unknown PPS-associated maculopathy status (Table 1), 19% (18/93) had macular abnormalities on examination. These macular findings included but were not limited to drusen (3 patients), pigmentary changes (3 patients), epiretinal membrane/macular pucker (4 patients), macular hole (1 patient), subretinal fluid (1 patient), and macular atrophy (1 patient). The median duration of exposure to PPS in this cohort of 93 patients was 1 year (range 0–13 years). The cumulative exposure of PPS among the 93 patients was median 113 total grams (range 2–1371). The daily dose by body weight in these 93 patients was median 4 mg/kg (range 2–8). The median BMI of these 93 patients was 27 kg/mg^2^ (range 18–51).

**Figure 1:**
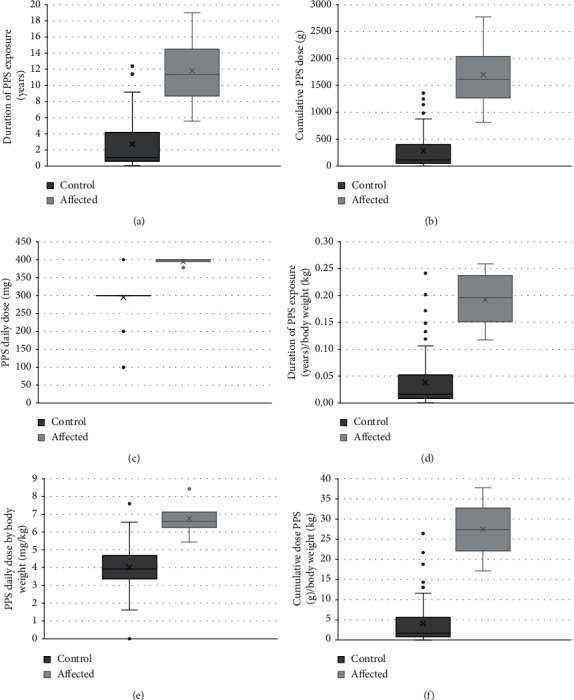
Patients affected by PPS maculopathy (*n* = 4) compared to control patients taking PPS without known maculopathy (*n* = 93). Duration of PPS exposure, also when considered by body weight, was higher in affected patients compared to controls. The daily dose of PPS was higher in affected patients compared to controls (middle row, left). Cumulative PPS exposure, also when considered by body weight, was higher in affected patients compared to controls (bottom row). Box plots show median center line in box, 1st and 3rd quartile edge of boxes, and end of lines showing minimum and maximum. Dots outside of the bars represent outliers. X in the boxes represents the mean. *P* values are results from two-tailed *t*-test. (a) *P*=0.05. (b) *P*=0.04. (c) *P*=6.0*e* − 7. (d) *P*=0.02. (e) *P*=0.02. (f) *P*=0.01.

Statistical comparison of patients affected by PPS maculopathy (*n* = 4) compared to control patients taking PPS without known maculopathy (*n* = 93) shows the duration of PPS exposure which, when considered by body weight, was higher in affected patients compared to controls. The daily dose of PPS was higher in affected patients compared to controls. Cumulative PPS exposure, also when considered by body weight, was higher in affected patients compared to controls (Figure 1).

Among the patients with possible PPS-associated maculopathy, detailed clinical history is provided.

**Figure 2:**
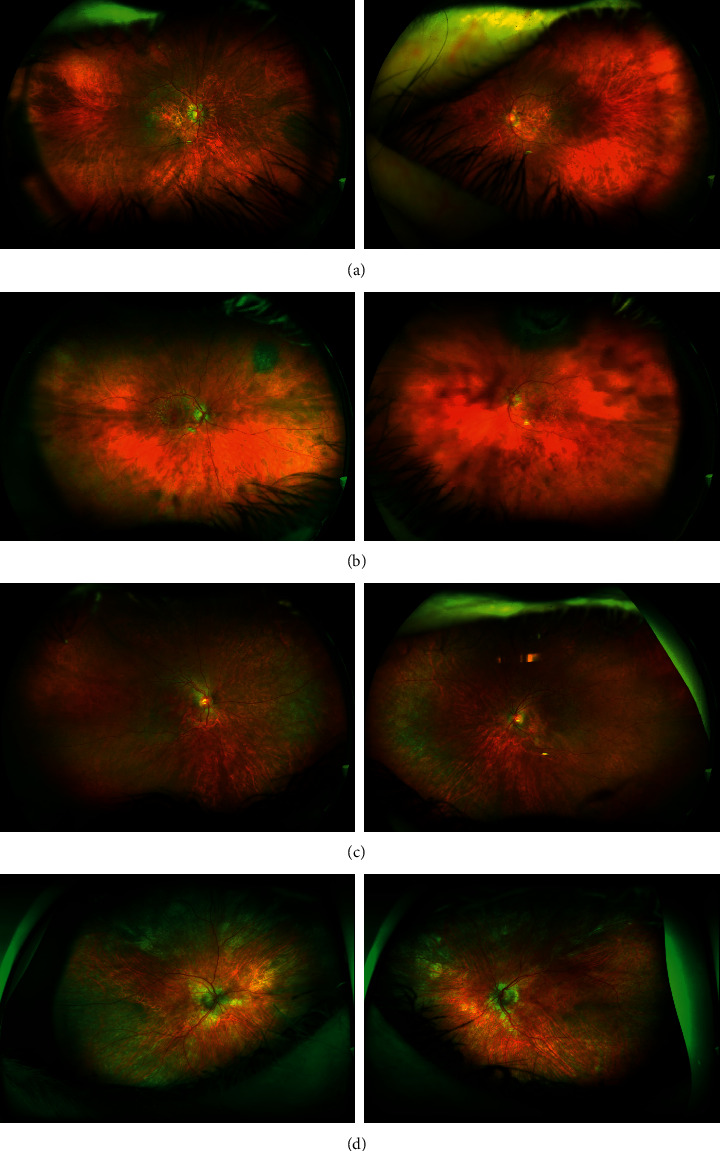
Ultrawidefield color fundus imaging of the retina in patient 1 (a), patient 2 (b), patient 3 (c), and patient 4 (d) demonstrates areas of macular atrophy and subtle pigmentary changes in both eyes. Patient 1 has a nevus in the right eye nasally, and patient 2 has a nevus in the right eye superonasally and a choroidal melanoma in the left eye superiorly. Patient 3 has reticular degeneration in the peripheral retina in both eyes.

**Figure 3:**
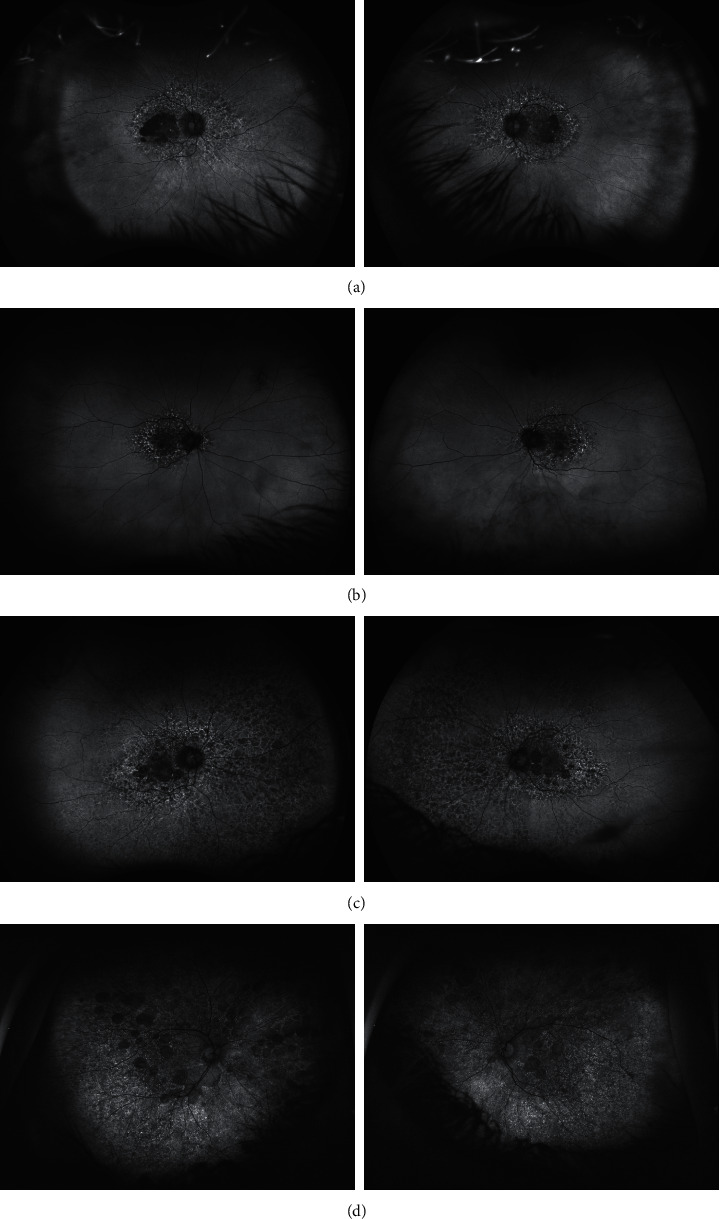
Ultrawidefield fundus autofluorescence imaging demonstrates lobules of absent autofluorescence in the macula of both eyes in patient 1 (a), patient 2 (b), patient 3 (c), and patient 4 (d). These lobules of macular atrophy are surrounded by a larger area of hyper- and hypoautofluorescent changes in the posterior pole of both eyes involving the macula and extending nasally beyond the optic nerve in each case. Patient 3 (c) has extensive peripheral retinal autofluorescence changes.

**Figure 4:**
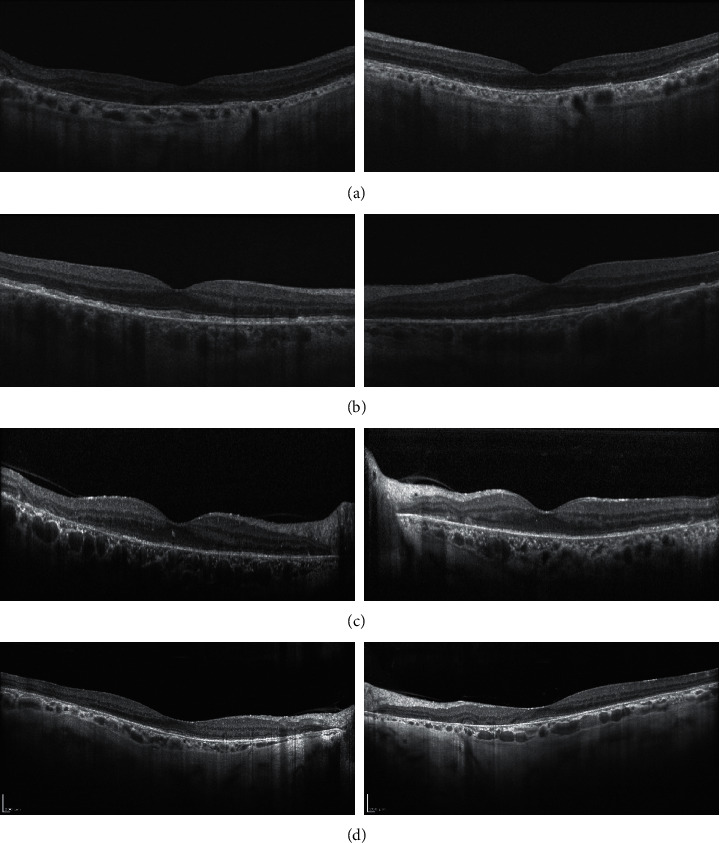
Spectral domain optical coherence tomography of the foveal center in patient 1 (a), patient 2 (b), patient 3 (c), and patient 4 (d) demonstrates attenuation of outer retinal lamination and retinal thinning. There is a diffuse loss of outer retinal reflectivity at the level of the RPE, ellipsoid zone (aka IS/OS junction), and an external limiting membrane. There are areas of increased choroidal transmission coincident with areas of RPE loss and disorganization of retinal layers.

**Figure 5:**
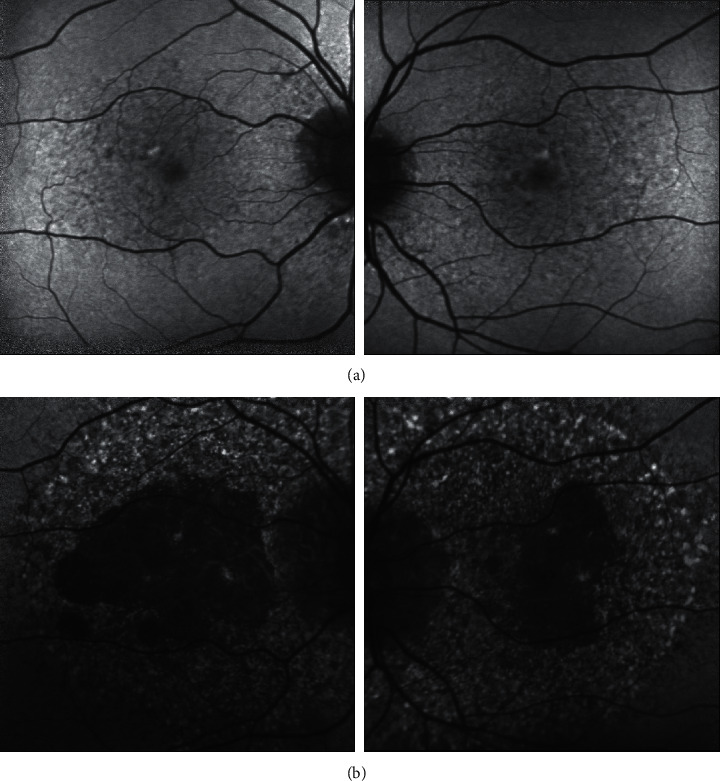
Progression of RPE macular atrophy. Images from patient 1 from July 2015 (20/40 OU) demonstrate a mottled pattern of autofluorescence (a). By October 2019 (OD: CF, OS: 20/50), the images show late-stage changes consisting of numerous large lobules of hypoautofluorescence in both eyes of patient 1 (b). These lobules of absent autofluorescence correspond to the lobules of macular atrophy seen on examination and on color fundus imaging (Figure 2). There is a broader area of speckled hyper- and hypoautofluorescent abnormality involving the entire macula.

Case 1 is a 67-year-old woman with a history of macular disease attributed to nonneovascular age-related macular degeneration with atypical features. She was diagnosed with interstitial cystitis about 20 years ago. She took PPS for 19 years at 400 mg daily and reports good control of her bladder symptoms. She reports slow degradation of her visual acuity in both eyes over the past few years. Since her last visit 6 months earlier, her right eye had become particularly blurred and was measured at counting fingers, while her left eye was 20/50. She was diagnosed with PPS maculopathy and PPS was stopped. One month later, her macular exam and imaging had not changed, but her visual acuity improved to 20/60 OD and 20/20 OS after stopping PPS. The ultrawidefield fundus image of the eyes is seen in Figure 2(a), fundus autofluorescence imaging is seen in Figure 3(a), and OCT images of the macula were seen in Figure 4(a). The progression of her macular disease over four years is depicted in Figure 5.

Case 2 is a 70-year-old woman with a history of macular disease attributed to atypical nonneovascular age-related macular degeneration. She also has a history of interstitial cystitis for the past 13 years. She has taken PPS to control her bladder pain successfully for 10 years at 400 mg daily. She stopped the medication about 2 years prior to her last ophthalmic presentation. She was seen by a retina specialist with expertise in ocular oncology for choroidal melanoma in the OS which was treated with proton beam therapy 3 months earlier. The tumor thickness appeared slightly reduced after treatment with stable borders and no evidence of radiation retinopathy was observed on examination. Her visual acuity was 20/25 OD and 20/50 OS at the time of diagnosis of PPS maculopathy which is unchanged from prior examinations. The ultrawidefield fundus image of the eyes is seen in Figure 2(b), fundus autofluorescence imaging is seen in Figure 3(b), and OCT images of the macula are seen in Figure 4(b).

Case 3 is an 81-year-old woman with a history of macular disease attributed to atypical nonneovascular age-related macular degeneration. She presented for a second opinion after seeing another retina specialist in the community who was concerned about inherited retinal disease due to the atypical findings in the macula. She has a history of interstitial cystitis diagnosed 6 years ago. She has taken PPS 400 mg daily for 6 years with good control of her bladder disease. She reports steadily declining vision in spite of cataract extraction in both eyes approximately one year ago. Her visual acuity at the time of PPS maculopathy diagnosis was counting fingers in both eyes. She stopped PPS and 6 months later her visual acuity remained stable at counting fingers with no subjective improvement. The ultrawidefield fundus image of the eyes is seen in Figure 2(c), fundus autofluorescence imaging is seen in Figure 3(c), and OCT images of the macula are seen in Figure 4(c).

Case 4 is a 74-year-old female with a history of pseudophakia who presented for a routine annual eye exam where a visual field showed a bitemporal pattern of loss suggesting a lesion on the optic chiasm. Magnetic resonance imaging of the brain showed a normal pituitary gland. Upon referral to a specialist, she was found to have diffuse retinopathy. She has a history of interstitial cystitis for the past 15 years for which she has taken PPS to control her bladder pain successfully for 13 years at 400 mg daily for the first 10 years, 300 mg daily for the final 3 years, and 100 mg daily for a final month. She reports some difficulty adjusting to light but denies blurry vision, pain, floaters, or photopsias. Her visual acuity was 20/40 OD and 20/40 OS at the time of diagnosis of PPS maculopathy. The ultrawidefield fundus image of the eyes is seen in Figure 2(d), fundus autofluorescence imaging is seen in Figure 3(d), and OCT images of the macula are seen in Figure 4(d).

## 4. Discussion

The mechanism of PPS-associated maculopathy is unclear, but several theories have been proposed. In IC, PPS acts as a GAG and coats the bladder epithelium in order to prevent irritants from reaching the underlying interstitium [8, 9]. One mechanism that we hypothesize is that circulating PPS may disrupt or deposit in the underlying Bruch's membrane of the retinal pigmented epithelium (RPE) and photoreceptor cells (PC). Bruch's membrane normally acts as a barrier between choroidal blood flow and the RPE [10]. Thickening of the membrane from increased deposition of GAG residues could lead to accumulation of lipofuscin granules within the RPE and PCs and subsequent cell death, due to alteration in the exchange of waste products between the choroidal blood flow and the RPE and PCs. This would be similar to the proposed mechanism in which dry age-related macular degeneration occurs, with protein and lipids accumulating in Bruch's membrane rather than GAG residues [11]. In the original market studies of pharmacodynamics of PPS, there was no evidence of distribution or accumulation of PPS in the eye [9]. However, these were short-term studies and identified patients with PPS-associated maculopathy appear to have had long-term exposure to PPS, ranging from a cumulative exposure of 440 g over 3 years to 4310 g over 20 years [3].

Other possible mechanisms for PPS-associated maculopathy have been proposed. In a letter to the editor of the original case report linking PPS exposure to pigmentary maculopathy [2], inhibition of the FGF pathway was implicated [12]. PPS has been reported to inhibit FGF1, FGF2, and FGF4 [13–15]. In zebrafish, it has been demonstrated that FGF signaling is required for maintenance of the retinal photoreceptor cells with inducible inhibition of FGF signaling causing progressive photoreceptor degeneration and disorganization of retinal tissue [16]. PPS has also been shown to inhibit DNA synthesis and cell migration of RPE cells in vitro [17]. PPS may impair RPE cell homeostasis through these mechanisms resulting in pigmentary changes and macular atrophy.

The screen for patients with PPS exposure history yielded 316 charts. After applying exclusion criteria, data of 216 charts were included for analysis. 96 of the charts had documented eye exams, 24 of which had retinal imaging done. Despite the original case series being published in 2018 [2], the majority of patients at risk had not received appropriate eye exams to assess for maculopathy.

Here, we present four cases of pigmentary maculopathy associated with PPS exposure identified after EMR screening of patients exposed to PPS. Among these 4 cases, the mean total exposure by duration was 12 years or 142 months and the average cumulative dose was 1701 g, indicating that long-term exposure to PPS is likely to be associated with maculopathy. This is similar to previous publications reporting an average cumulative dose of either 2202 g over an average of 186 months [2] or 1610 g over an average of 174 months [3]. The lowest reported total exposure to PPS-associated with maculopathy occurred over 36 months at a cumulative dose of 440 g [3]. Consistent with prior reports [2, 4, 5], macular atrophy was observed with relative preservation of central visual acuity in many patients, but severe vision loss from foveal involvement can occur, as seen with patient 3. Patient 1 lost vision to the counting fingers level in her right eye but after stopping the medication returned nearly to her baseline visual acuity of 20/60 one month later. This suggests that retinal toxicity may be reversible to some degree in select circumstances; however, it is unclear if this is a causative relationship. Cessation of PPS in cases of suspected retinal toxicity is recommended. This was done in all 4 cases identified in our report.

There are several limitations to our study, mostly due to the retrospective design. First, the true incidence of PPS-associated maculopathy cannot be determined based on our study. We identified 4 cases of PPS-associated maculopathy based on retinal imaging information that was available in a small subgroup of patients who were seen by a retinal specialist. Most patients in our study population did not see an ophthalmologist or have macular imaging at the study center. Since visual acuity may not be affected in PPS-associated maculopathy until later stages, there may be additional undiagnosed cases of PPS-associated maculopathy in our study population. Thus, all patients on PPS may benefit from a screening eye examination and macular imaging. Nonetheless, the fundus autofluorescence changes noted in all four cases showed changes similar to what has been reported for PPS-associated maculopathy. A screen of the electronic medical record is effective in identifying patients prior to severe vision loss. Use of EMR can easily identify those who currently take PPS or those with past exposure. However, it is limited in its ability to search for those with long-term exposure to PPS without individual medication history review. This is highlighted by the fact that in this report those with eye exams have an average of 1-year exposure to the drug. The lowest reported total exposure to PPS associated and development of a maculopathy occurred over 36 months at a cumulative dose of 440 g [3]; therefore, it is still useful to use EMR as a tool to identify those actively taking PPS for monitoring with routine eye exams and imaging, prior to the development of vision loss.

Despite these limitations, this study contributes to our knowledge of PPS-associated toxicity and presents additional cases to the growing body of literature on the development of maculopathy in the context of long-term PPS exposure. Furthermore, we show additional evidence that vision loss can be severe and progress relatively rapidly. Since the association between this maculopathy and PPS may not be obvious without macular imaging and detailed medical history, our study findings support the importance of referring patients to long-term PPS therapy for regular screening eye exams and imaging.

## Figures and Tables

**Table 1:** Demographics and pentosan polysulfate sodium exposure history for patients who had an eye exam (*n* = 96) and patient with maculopathy from outside referral (*n* = 1).

	PPS patients without maculopathy (*n* = 93 patients)	PPS patients with maculopathy (*n* = 4 patients)
Age, mean, years (median, range)	63 (67, 22–94)	73 (72, 67–81)

Race, no. (%)		
White	69 (74)	3 (75)
Black	1 (1)	0 (0)
Asian/Indian	10 (11)	0 (0)
Middle Eastern	0 (0)	0 (0)
Hispanic	3 (3)	0 (0)
Native American	1 (1)	0 (0)
Unknown	8 (9)	1 (25)
Mixed	1 (1)	0 (0)

Sex, no. (%)		
Male	12 (13)	0 (0)
Female	81 (87)	4 (100)

BMI, mean, kg/m ^ 2 (median, range)	28 (27, 18–51)	23 (23, 21–25)

Interval from date of IC diagnosis until now, mean, years (median, range)	9 (8, 1–43)	14 (14, 6–20)

Duration of PPS use, mean, years (median, range)	3 (1, 0–13)	12 (11, 6–19)

PPS daily dose, mean, mg (median, range)	295 (30, 100–400)	394 (400, 378–400)

PPS daily dose by body weight, mean, mg/kg (median, range)	4 (4, 2–8)	7 (6, 5–8)

Cumulative PPS exposure to date, mean, g (median, range)	295 (113, 2–1371)	1701 (1607, 812–2776)

BCVA OD logarithmic mean (median, range)	0.19 (0.10, 0.00–0.88)	0.6 (0.30, 0.00–1.90)

Snellen equivalent mean (median, range)	20/30 (20/25, 20/20–20/150)	20/80 (20/40–20/1600)

BCVA OS logarithmic mean (median, range)	0.16 (0.10, 0.00–0.54)	0.7 (0.40, 0.10–1.90)

Snellen equivalent mean (median, range)	20/30 (20/25, 20/20–20/70)	20/100 (20/50, 20/25–20/1600)

^∗^Patient 4 changed daily doses during treatment period; 378 mg represents the equivalent daily dose during the treatment period.

**Table 2:** Detailed clinical information of the 4 identified cases of pentosan polysulfate sodium associated maculopathy. U = unknown and W = white^*∗*^.

Age (years)	67	70	81	74

Race	U	W	W	W

Gender	F	F	F	F

Interval from date of IC diagnosis until now (years)	20	13	6	15

Duration of PPS use (years)	19	10	5	13

PPS daily dose (mg)	400	400	400	378^*∗*^

PPS daily dose by body weight (mg/kg)	5	7	8	7

BMI (kg/m^2)	25	21	21	23

Cumulative PPS exposure to date (g)	2776	1567	812	1794

Age at eye exam	66	69	81	74

Cumulative PPS at time of eye exam (g)	2694	1567	812	1794

Reason for visit	Postoperative visit	Choroidal malignancy	Second opinion for atypical macula findings	Referral for visual field loss

BCVA OD logarithmic	0.3	0	1.9	0.3

Snellen equivalent	20/40	20/20	20/1600	20/40

BCVA OS logarithmic	0.5	0.1	1.9	0.3

Snellen equivalent	20/63	20/25	20/1600	20/40

Tobacco and drug use	Yes (unknown pack-years)	No	Yes (1 pack-year)	No

Comorbidities	Depression, anxiety, and arthritis	Hypertension	Hypertension	Ulcerative colitis postcolectomy 4 years ago, chronic kidney disease not requiring dialysis, anxiety, and hypothyroidism

## Data Availability

The results from chart review used to support the findings of this study are restricted by the UC Davis School of Medicine Institutional Review Board and Health Insurance Portability and Accountability Act (HIPAA) in order to protect patient privacy. Data are available from the corresponding author for researchers who meet the criteria for access to confidential data.
